# Centrosome Polarization in T Cells: A Task for Formins

**DOI:** 10.3389/fimmu.2013.00191

**Published:** 2013-07-11

**Authors:** Laura Andrés-Delgado, Olga M. Antón, Miguel Angel Alonso

**Affiliations:** ^1^Centro de Biología Molecular Severo Ochoa, Consejo Superior de Investigaciones Científicas and Universidad Autónoma de Madrid, Madrid, Spain

**Keywords:** T cells, formins, microtubule-organizing center, detyrosinated microtubules, tyrosine phosphorylation

## Abstract

T-cell antigen receptor (TCR) engagement triggers the rapid reorientation of the centrosome, which is associated with the secretory machinery, toward the immunological synapse (IS) for polarized protein trafficking. Recent evidence indicates that upon TCR triggering the INF2 formin, together with the formins DIA1 and FMNL1, promotes the formation of a specialized array of stable detyrosinated MTs that breaks the symmetrical organization of the T-cell microtubule (MT) cytoskeleton. The detyrosinated MT array and TCR-induced tyrosine phosphorylation should coincide for centrosome polarization. We propose that the pushing forces produced by the detyrosinated MT array, which modify the position of the centrosome, in concert with Src kinase dependent TCR signaling, which provide the reference frame with respect to which the centrosome reorients, result in the repositioning of the centrosome to the IS.

## Introduction

T cells polarize at the cell-to-cell contact in response to appropriate antigens presented by an antigen-presenting cell (APC), forming a surface subdomain known as the immunological synapse (IS) (1, 2). The T-cell antigen receptor (TCR), adhesion molecules and other membrane receptors, signaling molecules, such as the tyrosine kinase Lck, and cytoskeletal proteins, such as actin, concentrate at the IS. Polarization of the T-cell surface is accompanied by reorganization of the microtubule (MT) cytoskeleton and reorientation of the centrosome, the major MT-organizing center (MTOC), to face the IS. The reorientation of the MTOC, which is one of the hallmarks of T-cell polarization, is required for normal signaling through the TCR (3) and polarization of the secretory apparatus to the IS to facilitate T-cell effector functions (4, 5).

Pioneering work established that the MT cytoskeleton is essential for MTOC polarization in T cells as this process is inhibited by nocodazole, which completely disrupts the MT network (6–8). Most mammalian cells have two subsets of MTs: dynamic MTs, with short half-lives, and stable MTs, which are of longer duration. The observation that the treatment of T cells with taxol, an MT-stabilizing drug, does not interfere with MTOC polarization indicates that the dynamic MT pool is not important for this process (9). Despite the time that has passed since then and the importance of the process of MTOC reorientation to T-cell function, little progress has been made toward determining the nature of the tubulin modifications required for MT cytoskeleton remodeling, the mechanism by which MTs are stabilized after TCR engagement, the identification of the machinery involved, or the exact role of MTs in MTOC reorientation.

The tyrosine kinases Lck, Fyn, and ZAP-70 (10–12), novel and atypical protein kinase C isoforms (13, 14), the (−) end MT motor dynein (3, 15, 16), and diacylglycerol accumulation at the IS (16) are essential for MTOC polarization. Recent findings indicate that at least three formins collaborate to generate a specialized array of MTs that mediates the process of MTOC polarization (17). Herein we have integrated recent results concerning the role of formins in MT remodeling with previous observations, including the participation of the MT cytoskeleton and the requirement for Src kinase dependent TCR signaling in MTOC reorientation. We argue for a new framework for the long-unresolved matter of the role of MTs in MTOC polarization, and pose important questions about how the polarization of the MTOC takes place.

## Formins in T Cells

Most formins are direct effectors of Rho-family GTPases (18). Unlike the actin-related protein 2/3 (Arp2/3) complex, which forms branched filaments, formins generate linear filaments (19). The defining feature of all formin proteins is the ∼400-amino acid formin homology (FH) 2, which mediates actin assembly. In humans, formins are represented by 15 members that are classified into seven groups by phylogenetic analysis of the FH2 domains. Formins are known to modulate a number of intracellular processes, such as endosome motility, MT stabilization, and cytokinesis (20).

**Figure 1:**
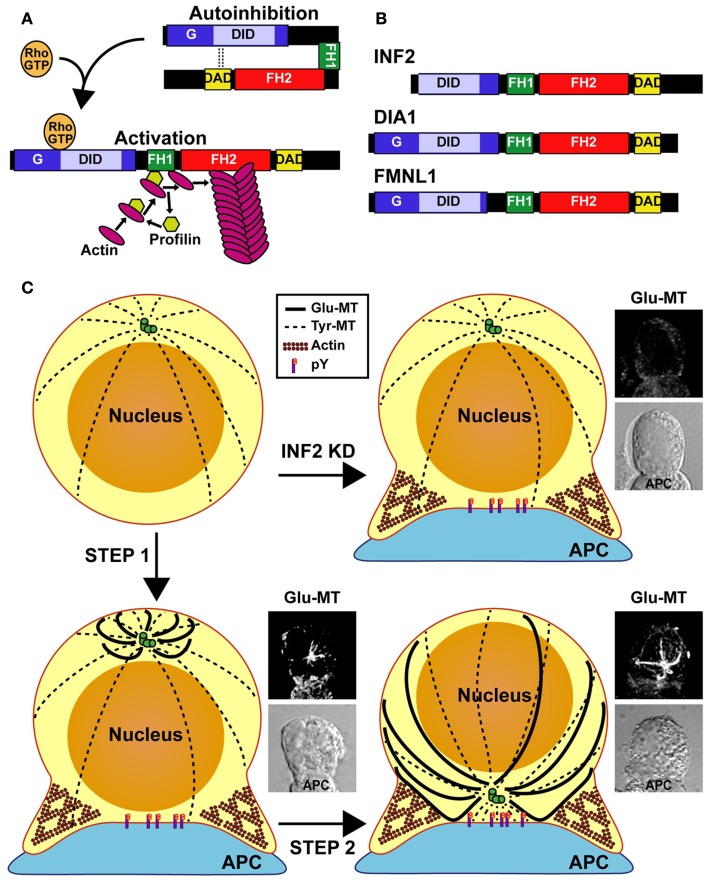
**Model of MTOC repositioning in T cells**. **(A)** Regulation of the diaphanous-related formins. The autoinhibitory effect of the DID-DAD interaction is released through binding of a specific Rho-family GTPase in its active GTP-loaded form. In the open conformation of formins, the FH1 domain recruits profilin that, in turn, brings actin monomers to the proximity of the FH2 domain for actin polymerization. **(B)** Domain organization of INF2, DIA1, and FMNL1. The molecules are not drawn to scale. **(C)** Model of T repositioning. The MT array is organized radially in resting T cells. After the TCR recognizes an antigen presented by an antigen-presenting cell (APC), INF2 and other formins promote the formation of a Glu-MT array in the T cell, which pushes the MTOC (Step 1). Simultaneously, Src kinase dependent TCR signaling (phosphotyrosine, pY) directs the movement of the MTOC toward the IS. Finally, Glu-MTs would maintain the MTOC at the IS and could be used for membrane trafficking from the (+) MT ends, situated at the posterior part of the cell, toward MTOC-located (−) MT ends (Step 2). In the absence of INF2 (INF2 KD), no Glu-MTs are formed and the MTOC cannot move even though Fyn and Lck-dependent tyrosine phosphorylation takes place.

Diaphanous-related formins such as mDia 1–3 have an autoregulatory domain at their carboxyl half, known as the diaphanous autoregulatory domain (DAD), which is separated by FH1 and FH2 domains from the diaphanous inhibitory domain (DID) present at the amino-terminal half. A short amino-terminal extension (G) precedes the DID. The DAD interacts with the DID to close the diaphanous-related formin molecule and maintain it in an inactive state. The binding of the effector Rho GTPase to the GTPase-binding domain, which encompasses the G extension and the amino-terminal part of the DID, regulates diaphanous-related formins by releasing the DID-DAD interaction and opening up the molecule (Figure 1A) (21). Unlike DIA1 (the human ortholog of mDia1) and FMNL1, INF2 lacks the amino-terminal extension that includes the G region, and contains a Wasp-homology 2 (WH2) sequence within its DAD (Figure 1B). The presence of the WH2 sequence makes INF2 able not only to nucleate actin polymerization but also to depolymerize actin filaments *in vitro* (22).

The formins DIA1, FMNL1, and INF2 are so far the most extensively characterized formins in T cells. In addition to localizing to the plasma membrane, DIA1, FMNL1, and INF2 distribute along MTs and at the MTOC in resting T cells. mDia1 expression is induced during T-cell activation and regulates cell migration (23). Consistently, T cells from mDia1 knockout mice were defective in migration and proliferation in response to chemotactic and proliferative stimuli, respectively (24, 25). Although a large pool of DIA1, FMNL1, and INF2 localize with filamentous actin at the IS, and despite the best characterized function of formins being the nucleation of actin filaments, the three formins are not necessary for actin polymerization at the IS (17, 26). The Arp2/3 complex controls this process (26).

DIA1 and FMNL1 were found to be essential for MTOC reorientation in Jurkat cells and primary T lymphocytes (26). Although a recent report indicates that lytic granule secretion is not necessarily coupled to MTOC polarization (27), the knockdown of DIA1 or FMNL1 reduced cytolytic activity of primary human CD8^+^ T cells (26). More recently, INF2 has been demonstrated to be necessary for MTOC reorientation in primary T cells and in Jurkat cells (17). Deletion analysis identified the FH2 as the INF2 domain responsible for MTOC repositioning. Importantly, the actin polymerization activity of the FH2 domain was not essential for mediating this process, which is consistent with observations showing that actin dynamics are not necessary for MTOC polarization (17, 28). The involvement of DIA1, FMNL1, and INF2 indicates that these and probably other formins are necessary for MTOC repositioning to take place.

## TCR Engagement Induces Formation of an Array of Specialized Detyrosinated MTs

Dynamic MTs are locally stabilized during many morphogenetic events, including cell migration, muscle development, neurite outgrowth, and epithelial polarization (29). Localized MT stabilization results from the capping of MT plus-ends to prevent subunit exchange (30). One of the posttranslational modifications of tubulin in stabilized MTs is the detyrosination of the carboxyl-terminal Tyr residue of α-tubulin and the subsequent exposure of the adjacent glutamate residue, generating Glu-MTs (31). In addition, MTs undergo other types of modification, including acetylation, polyglutamylation, polyglycylation, phosphorylation, and palmitoylation (32).

T-cell antigen receptor engagement produces the rapid formation of a specialized array of stable Glu-MTs in Jurkat cells and in primary T cells (17). Almost all signal transduction from the TCR is believed to occur through tyrosine phosphorylation. However, it is of particular note that Src-family tyrosine phosphorylation is not involved in Glu-MT formation in T cells since this process occurred equally well in the presence of an inhibitor of Src-family kinases (17). This finding is consistent with previous observations that Glu-MT formation is not affected in fibroblasts from triple Src, Yes, Fyn knockout mice (33).

## INF2, DIA1, and FMNL1 Promote the Formation of Glu-MTs Necessary for MTOC Reorientation

In fibroblasts, the GTPase RhoA controls the formation of stable Glu-MTs through its effector mDia1 (34). The FH2 domain of mDia2 is able to promote formation of Glu-MTs independently of its actin polymerization activity. The FH2 domain of mDia2 inhibits the polymerization and depolymerization rates of MTs probably by the formation of a multiprotein complex at the MT ends. This activity may contribute to MT stabilization and, subsequently, to MT detyrosination by a still unknown mechanism (35). Since the FH2 domain is highly conserved in formins (36, 37), other formins in addition to mDia1-2 are probably also able to promote the formation of Glu-MTs.

Casein kinase I delta (8) and stathmin (38) control MT dynamics and MTOC repositioning in T cells. Similar to the requirement for INF2 for polarizing MTOC to the IS, the formation of the Glu-MT array induced after T-cell engagement required the expression of INF2. Moreover, as is the case of mDia2 in fibroblasts (35), the formation of Glu-MTs by INF2 in T cells occurred independently of INF2 actin polymerization activity. Importantly, pharmacological treatment of the cells with concentrations of taxol that stabilize MTs and induce Glu-MTs but do not completely block MT dynamics (39) corrected the defect of Glu-MT formation and MTOC repositioning found in INF2 knockdown cells. DIA1 and FMNL1, which are also essential for MTOC reorientation, were found to be necessary for the formation of the Glu-MT array in T cells (17). Together, these observations indicate that the formins INF2, DIA1, and FMNL1 promote the formation of a Glu-MT array that is crucial to MTOC polarization.

The DID of INF2 interacts with the DAD of mDia1, the interaction inhibiting actin polymerization by mDia *in vitro* (40). Therefore, it is conceivable that INF2 forms a complex with other formins, and probably with other proteins. This complex caps the MT ends and leads to MT stabilization. As was seen with mDia2 (35), mDia1 and INF2 bind MTs through their FH2 domain (41). Differences between these three formins in their interactions with MTs and actin, regulation by MTs and actin, and effect on MT bundling (41) suggest that they could play specific complementary roles in regulating MT function and structure.

## Cdc42 and Rac Regulates Glu-MT Formation and MTOC Reorientation

Formins are primarily regulated through interactions with Rho-family GTPases. INF2 associates with Cdc42 and Rac1 (42, 43), FMNL1 with RhoA and Rac1 (26, 44), and mDia1 with Rho (45). Expression of a dominant negative form of Cdc42, which sequesters Cdc42 effectors, indicated a role for Cdc42 in MTOC polarization (46). However, conflicting results have been obtained in Cdc42 knockdown experiments using siRNA interference (17, 26), whereby the Cdc42 effectors were left free to interact with other protein partners, probably due to different silencing efficiencies. Rac1 was also involved in regulating MTOC reorientation as Rac1 knockdown reduced the percentage of cells with MTOC reoriented (17, 26). Consistent with the binding of Rac1 and Cdc42 to INF2 (43) and with the involvement of INF2 in Glu-MT formation and MTOC repositioning, the silencing of either GTPase impaired both processes. These observations suggest that Cdc42 and Rac1 exploit formin effectors to control the formation of stable Glu-MTs necessary for MTOC repositioning.

## Glu-MTs and TCR-Induced Tyrosine Phosphorylation are Simultaneously Required for MTOC Reorientation

Although Glu-MT formation in T cells is insensitive to Src-family tyrosine kinase inhibition, the MTOC reorients to the IS only when Glu-MT formation concurs with Src kinase dependent signaling (17). In other words, TCR-induced tyrosine phosphorylation at the IS provides the reference frame with respect to which the MTOC reorients. This finding is consistent with previous results showing that the tyrosine kinases Lck, Fyn, and ZAP-70 are important for MTOC reorientation (10–12).

Glu-MTs appear to control MTOC displacement although the directionality of the movement to the IS is regulated by TCR signaling. In the absence of Lck the MTOC polarized toward the IS although it was unable to dock at the IS, whereas in the absence of Fyn or of both Lck and Fyn MTOC polarization was completely impaired and the MTOC remained far away from the IS (12, 47). Therefore, MTOC polarization appears to be a multistep process in which stable Glu-MTs mediate MTOC movement and polarized TCR signaling controls its position. Once the MTOC has docked at the IS, Glu-MTs, which are seen as long MTs, attach to distal sites of the plasma membrane relative to the IS. Some of these Glu-MTs touch the IS and then bend backwards, extending to the posterior region of the cell (17). Consistent with these observations, we know that long MTs curve past the LFA1-enriched ring of the IS contacting the plasma membrane *en route* to their minus ends at the MTOC in cytotoxic T lymphocytes conjugated to target cells (48). Therefore, in addition to their role in MTOC positioning, curved Glu-MTs contacting the IS could serve as tracks for membrane trafficking from the (+) MT ends, situated at the posterior part of the cell, toward MTOC-located (−) MT ends, which are situated at the IS. Eventually, cargo would be delivered directly since the transport vesicles might encounter the IS when traveling along the curved Glu-MTs before they ever reach the MTOC (49, 50). In contrast, dynamic MTs with their (+) end oriented toward the IS mediate vesicular transport in a (−) to (+) MT end direction, as has been recently observed for the TCR (51).

## How do Glu-MTs Direct MTOC Reorientation?

In cell types, such as fibroblasts, that have a radial MT organization and that polarize during cell migration extended on the substrate the MTOC is maintained at the cell center. During fibroblast polarization in wound-healing assays, the MTOC remains stationary whereas the nucleus moves backwards, resulting in MTOC orientating toward the wound edge (52). In small spherical cells with a radial MT organization, such as resting T lymphocytes, the nucleus is located at the cell center, occupying most of the cell’s interior and leaving little space for organelles between the nucleus and the plasma membrane. Therefore, unlike fibroblasts, the MTOC is constitutively asymmetrically positioned in T lymphocytes due to space constraints. Dynamic MTs tend to center the MTOC via a geometric action: if the MTOC is displaced from the center, more MTs will contact the cortex on the nearer side because it takes less time for MTs to reach the closer part of the cortex. This leads to a net force pushing the MTOC toward the cell center (53). Therefore, the asymmetric position of the MTOC in resting T cells cannot be maintained by dynamic MTs. Instead, stable MTs, which generate opposing pushing forces, are required to maintain the MTOC off-cell center. It is of note that stable acetylated MTs are found in resting T lymphocytes where, as for the bulk of MTs, they are radially organized. It is therefore conceivable that the acetylated MT pool or other type of stable MT pool is responsible for maintaining the asymmetric position of the MTOC in resting T cells.

In principle, there are two possibilities for MTOC polarization after TCR recognition of an antigen presented by an APC. One is that the T cell rotates until the MTOC faces the IS. However, it is difficult to envision how the T cell can rotate without disengaging from the APC. Indeed, no such movement has been observed by videomicroscopy. A more likely scenario is that the MTOC moves toward the IS. For net movement, the MTOC requires asymmetrical pushing and/or pulling forces. It has been observed that after TCR engagement, but before reorientation is complete, Glu-MTs organize with their MT plus-ends directed toward the plasma membrane region opposite the IS. This event breaks the symmetrical radial organization of MTs seen in resting T cells and could produce the pushing forces required to displace the MTOC toward the IS (Figure 1C). It is possible that the (−) end MT motor dynein could bind the MTs to displace the MTOC toward the IS by MT pulling (3, 15, 16).

It was proposed that posttranslational modifications of tubulin mark subpopulations of MTs and selectively affect downstream MT-based functions. In this way, the tubulin modifications would generate a “code” that can be read by MT-associated proteins in a manner analogous to that by which the “histone code” directs diverse chromatin functions (54). A major implication of the “tubulin code” is that posttranslational modifications influence the recruitment of protein complexes, which in turn contribute to MT-based functions. In this sense, the presence of the carboxyl-terminal Tyr residue of α-tubulin is crucial for MT interaction with plus-end-tracking proteins containing cytoskeleton-associated protein-glycine-rich (CAP-Gly) MT-binding domains (55, 56) and regulates kinesin-1 motor binding to MTs and the association of MTs with intermediate filaments (57–59). It is therefore plausible that the loss of the carboxyl-terminal Tyr residue in response to TCR triggering modulates the interaction of MTs with the specific protein machinery involved in the move of the MTOC toward the IS.

## Outstanding Questions

A large variety of molecules acting downstream of the TCR, such as Lck, Fyn, and ZAP-70 and some of their important substrates, such as the LAT and SLP-76 adapters, and Rho-family GTPases are required for MTOC repositioning in T cells. In addition, INF2, with the participation of at least two other formins, DIA1 and FMNL1, promotes independently of Src kinase dependent TCR signaling the formation of a specialized array of stable Glu-MTs that is essential for MTOC polarization. The questions arise as to: (1) how the TCR communicates with formins to promote the formation of the Glu-MT array, (2) how the different formins work together, and probably with other proteins, to promote the formation of the array, (3) how Glu-MTs move the MTOC, and (4) how Glu-MTs coordinate with TCR-induced Fyn and Lck signaling for polarized movement of the MTOC toward the IS.

## Conflict of Interest Statement

The authors declare that the research was conducted in the absence of any commercial or financial relationships that could be construed as a potential conflict of interest.
